# A CRISPR/Cas12a-Based System for Sensitive Detection of Antimicrobial-Resistant Genes in Carbapenem-Resistant *Enterobacterales*

**DOI:** 10.3390/bios14040194

**Published:** 2024-04-16

**Authors:** Jiyong Shin, Sei Rim Kim, Zifan Xie, Yong-Su Jin, Yi-Cheng Wang

**Affiliations:** 1 Department of Food Science and Human Nutrition, University of Illinois Urbana-Champaign, Urbana, IL 61801, USA; 2 Center for Digital Agriculture, University of Illinois Urbana-Champaign, Urbana, IL 61801, USA

**Keywords:** biosensing, fluorescence assay, food safety, public health, multi-drug-resistant bacteria

## Abstract

Antimicrobial-resistant (AMR) bacteria pose a significant global health threat, and bacteria that produce New Delhi metallo-β-lactamase (NDM) are particularly concerning due to their resistance to most β-lactam antibiotics, including carbapenems. The emergence and spread of NDM-producing genes in food-producing animals highlight the need for a fast and accurate method for detecting AMR bacteria. We therefore propose a PCR-coupled CRISPR/Cas12a-based fluorescence assay that can detect NDM-producing genes (bla_NDM_) in bacteria. Thanks to its designed gRNA, this CRISPR/Cas12a system was able to simultaneously cleave PCR amplicons and ssDNA-FQ reporters, generating fluorescence signals. Our method was found to be highly specific when tested against other foodborne pathogens that do not carry bla_NDM_ and also demonstrated an excellent capability to distinguish single-nucleotide polymorphism. In the case of bla_NDM_-_1_ carrying *E. coli*, the assay performed exceptionally well, with a detection limit of 2.7 × 10^0^ CFU/mL: 100 times better than conventional PCR with gel electrophoresis. Moreover, the developed assay detected AMR bacteria in food samples and exhibited enhanced performance compared to previously published real-time PCR assays. Thus, this novel PCR-coupled CRISPR/Cas12a-based fluorescence assay has considerable potential to improve current approaches to AMR gene detection and thereby contribute to mitigating the global threat of AMR.

## 1. Introduction

Antimicrobial resistance (AMR)—microorganisms’ ability to survive and grow in the presence of antibiotics or other antimicrobial substances designed to inhibit or kill them—is an increasingly urgent global public-health threat [1]. As antimicrobials become less effective or in some cases wholly ineffective against certain microorganisms, treating the resulting infections becomes extremely difficult. According to the Centers for Disease Control and Prevention [2], AMR was associated with approximately 5 million deaths globally in 2019, and more recently, the World Health Organization (WHO) listed AMR as one of the top 10 public-health issues facing the world [3]. Therefore, monitoring AMR is of immense importance.

Carbapenems are a class of beta-lactam antibiotics that can inhibit bacterial cell-wall synthesis and thus are effective against many aerobic and anaerobic Gram-positive and Gram-negative bacteria [4]. They are deemed last-resort antibiotics that can be used to treat multi-drug-resistant infections when microorganisms cannot be killed by other antibiotics [5]. However, due to the abuse and/or overuse of such antibiotics, the emergence of carbapenem-resistant *Enterobacterales* (CRE) is a serious issue worldwide and was recently listed as one of five urgent threats by the CDC [6]. More importantly, although the use of carbapenems to treat food-producing animals is restricted, the wide treatment of livestock with other beta-lactams such as cephalosporins, e.g., cefradine, ceftiofur, and cefotaxime, may promote carbapenem resistance [7]. In fact, CRE has been identified globally in food-producing animals as well as in wildlife and pets [8]. As the transmission of CRE from these and other environmental sources to humans is possible, it is a major public-health priority to monitor CRE, with the aim of preventing its further spread [9]. Accordingly, considerable efforts have been made to develop CRE- and other AMR-monitoring technologies and systems, especially for use in animal-based food-production settings [10].

CRE-detection methods include culture-based assays, immunochromatographic assays, matrix-assisted laser desorption ionization-time of flight mass spectrometry, and deoxyribonucleic acid (DNA)-based assays [11]. Among the DNA-based techniques, polymerase chain reaction (PCR), which detects CRE by monitoring bacterial DNA, is one of the most commonly used [12]. However, conventional PCR has its limits. It is a laborious multistep process that includes the use of gel electrophoresis for the visualization of results and has low sensitivity [12]. False positives may also be caused by non-specific amplification or primer dimers. More importantly, conventional PCR’s ability to discriminate single-nucleotide polymorphism (SNP) is also limited, leading to low specificity. Therefore, there is an unmet need to develop a highly sensitive, highly specific, and easy-to-use technique for CRE detection.

CRISPR is an acronym for clustered regularly interspaced short palindromic repeats, which were discovered in prokaryotes’ adaptive immune systems and enable them to defend against invasive elements [13]. Since its discovery in 2007, the system that comprises CRISPR and its associated nuclease, known as Cas, has been applied to various purposes, including both genome editing and pathogen detection [14]. CRISPR/Cas systems typically have two main components: a guide ribonucleic acid (gRNA) and a Cas protein, with the former being used to direct the latter’s nuclease to target nucleic acids. Different Cas nucleases isolated from various bacteria can achieve different cleaving functions. Commonly used Cas nucleases include Cas9, Cas12, and Cas13. Their detection methods vary due to inherent differences, particularly related to target nucleotides and trans-cleavage activity. For instance, Cas12 and Cas13 exhibit trans-cleavage activity, unlike Cas9, and Cas12 targets DNA, whereas Cas13 targets RNA [14].

CRISPR/Cas12a nuclease is widely utilized in nucleic-acid detection. However, it was also chosen for this study for two further reasons: (1) it recognizes DNA targets, and (2) it exhibits a trans-cleavage effect, i.e., collateral cleavage of untargeted sequences, in addition to cleavage of the target sequence (cis-cleavage) [15]. In the presence of the target gene, Cas12a recognizes the protospacer-adjacent motif (PAM) sequence (TTTV, where V can be A, C, or G); binds to the target sequence via gRNA; and cleaves it [16]. This initiates untargeted trans-cleavage activity, and by leveraging such activity using probe-modified ssDNA, target recognition can be turned into a signal. Due to CRISPR/Cas systems’ ability to recognize nucleic acids, they have considerable potential to detect genes. In a comparative study of nucleic-acid detection methods, CRISPR/Cas12a-based detection was better able to detect SNP than conventional PCR, quantitative PCR (qPCR), recombinase polymerase amplification (RPA), or loop-mediated isothermal amplification (LAMP) [17]. Although few studies have achieved the highly specific and sensitive detection of AMR using CRISPR-based systems, little research has hitherto explored using such systems for CRE detection [18].

Accordingly, this paper proposes a new CRISPR/Cas12a-based detection assay coupled with PCR to sensitively detect CRE. Specifically, it was designed to detect the New Delhi metallo-β-lactamase (NDM) gene, one of the most problematic AMR genes, which encodes carbapenemases: beta-lactamase enzymes that can hydrolyze carbapenems [19]. In part, because it utilizes a CRISPR/Cas12a system in place of gel electrophoresis to identify the amplicon from PCR, our assay can attain excellent analytical performance marked by enhanced simplicity, sensitivity, and specificity. The specific binding of gRNA to the amplicon in the CRISPR/Cas system confirms the presence of the amplicon by the nucleotide sequence rather than by the amplicon’s size. This addresses important weaknesses of gel electrophoresis, i.e., it not only reduces the incidence of false positives but also enables SNP discrimination. The method’s real-world applicability was also evaluated by testing its performance with food samples. Accordingly, we believe that our proposed assay has excellent potential to improve monitoring for CRE and thereby contribute to mitigating the deadly global threat of AMR.

## 2. Experimental Section

### 2.1. Materials

Nuclease-free water and oligonucleotides including Hexachloro-fluorescein (HEX)-labeled single-stranded DNA-fluorophore-quencher (ssDNA-FQ) reporter, primer sets, dsDNA for mismatch analysis, and guide RNA were all purchased from Integrated DNA Technologies (IDT, Coralville, IA, USA). All the nucleotide sequences used are listed in Table 1. NEBuffer 2.1, EnGen Lba Cas12a, HiScribe™ T7 Quick High Yield RNA Synthesis Kits, DNase I, and RNase inhibitor were purchased from New England Biolabs (Ipswich, MA, USA), and buffer peptone water (BPW) and phosphate buffer saline (PBS) were from MilliporeSigma (Burlington, MA, USA). QIAquick PCR purification kits and an RNeasy MinElute cleanup kit were purchased from Qiagen (Germantown, MD, USA), and PrimeSTAR HS DNA polymerase for PCR was from Takara Bio USA (San Jose, CA, USA).

### 2.2. Bacterial Culturing and Genomic-DNA Extraction

Of the five bacterial strains used in this study, one—bla_NDM-1_ carrying *Escherichia coli* (*E. coli*, ATCC BAA-2471)—was purchased from ATCC (Manassas, VA, USA). The remaining four—*E. coli* O157:H7 (ATCC 43890), *Staphylococcus aureus* (*S. aureus*, ATCC 6583), *Listeria monocytogenes* (*L. monocytogenes*, ATCC 19112), and *Salmonella enterica* serovar Typhimurium (*S.* Typhimurium, ATCC 19585)—were provided by Dr. Michael Miller at the University of Illinois Urbana-Champaign. *L. monocytogenes* was inoculated in a brain-heart infusion medium (Teknova, Hollister, CA, USA), and all other strains were in nutrient media (BD, Franklin Lakes, NJ, USA), overnight at 37 °C. The resulting bacterial cultures were then diluted in 1× PBS, and DNA was extracted from them using a DNeasy blood and tissue kit (Qiagen, Germantown, MD, USA) according to the manufacturer’s instructions. The purity and concentration of the extracted DNA samples were measured using a nanodrop spectrophotometer (Thermo Fisher Scientific, Waltham, MA, USA). All DNA samples were then stored at −20 °C until analysis.

### 2.3. Preparation of gRNA

All the gRNA sequences we used in gRNA selection were produced by in vitro transcription. Template DNA including the gRNA sequences and T7 RNA polymerase promotor was amplified via PCR. Specifically, PCR was performed via (1) predenaturation at 98 °C for 10 s; (2) 35 cycles each consisting of denaturation at 98 °C for 10 s, annealing at 54 °C for 20 s, and extension at 72 °C for 30 s; and (3) a final extension at 72 °C for 5 min. The PCR products were then purified using the above-mentioned QIAquick PCR purification kit according to its manufacturer’s protocol. A total of 1.5 µg of each PCR product was transcribed in vitro overnight at 37 °C with the HiScribe™ T7 Quick High Yield RNA Synthesis Kit. DNase I was then added, and the resulting mixture was incubated at 37 °C for 30 min to remove the template DNA. The in vitro-transcribed RNA was purified using the RNeasy MinElute Cleanup kit according to the manufacturer’s protocol. The purity and concentration of the RNA samples were then measured using the nanodrop spectrophotometer, and the samples were stored at −80 °C until further analysis.

### 2.4. Antimicrobial Gene (bla_NDM-targeted_) Preparation

The full NDM-targeted gene (bla_NDM-targeted_, 813 bp) was amplified via PCR from the DNA genome extract of bla_NDM-1_-carrying *E. coli*. Specifically, PCR was performed on a 25 µL mixture containing PCR mastermix, 400 nM of each primer, and 10 ng of DNA template. In this case, the PCR process consisted of (1) predenaturation at 98 °C for 5 min; (2) 30 cycles each consisting of denaturation at 98 °C for 10 s, annealing at 58 °C for 5 s, and extension at 72 °C for 1 min; and (3) a final extension at 72 °C for 5 min. The PCR products were purified using the QIAquick PCR purification kit according to the manufacturer’s instructions. The amplified bla_NDM-targeted_ genes were analyzed using gel electrophoresis (at 100 V applied potential for 50 min) with 1.4% agarose gel (Thermo Fisher Scientific, Waltham, MA, USA) containing SYBR Safe (NEB, Ipswich, MA, USA) in 0.5 × Tris-acetate-EDTA (TAE) buffer (Thermo Fisher Scientific, Waltham, MA, USA).

### 2.5. CRISPR/Cas12a Fluorescence Assay

All extracted bacterial DNA samples were amplified using PCR under the same conditions described in Section 2.4. Each Cas12a reaction contained 100 nM of Cas12a, 100 nM of gRNA, 100 nM of ssDNA-FQ reporter, 12 µL of PCR product, and 9 µL of NEBuffer 2.1. The total volume of the assay was then adjusted to 90 µL using nuclease-free water, and each sample’s fluorescence was measured for 30 min at 37 °C. To facilitate comparisons between samples within each experiment, emission was read in relative fluorescence units (RFU). To allow comparison between our technique and conventional PCR, 12 µL of PCR product was also analyzed using gel electrophoresis with the same parameters described in Section 2.4.

### 2.6. Mismatch Analysis

To determine whether our assay could distinguish target DNA with a single-nucleotide mismatch, we used 28 bp dsDNA target sequences including the PAM sequence and the gRNA binding site. The assigned mismatch positions were the third position of the PAM sequence and the second, fourth, sixth, and eighth positions on the upper DNA target strand. To test the single-base resolution of the assay, we performed the same CRISPR/Cas12a fluorometric assay described in Section 2.5.

### 2.7. Detection of the Target AMR Gene in Real Food Samples

Our evaluation of the target AMR gene in real food followed previously published methods [20,21], with slight modifications. Pre-cut chicken was purchased from a local market (Champaign, IL, USA) and stored at 4 °C until it was needed for our experiments. Then, it was placed in a sterile bag with BPW and homogenized for 3 min. The chicken rinse was aliquoted to sterile tubes, which were then inoculated with CRE strains to create eight solutions with final CRE concentrations ranging from 10^0^ to 10^7^ CFU/mL. Two negative control sample groups were also prepared: one without bacteria and the other containing 10^7^ CFU/mL of *E. coli* O157:H7, which lacked the target AMR gene. Bacterial DNA was extracted from all samples using the DNeasy blood and tissue kit according to the manufacturer’s protocol and stored at −20 °C until further analysis.

### 2.8. Data Collection and Processing

All fluorescence measurements were performed using an M5 fluorescence plate reader (SpectraMax, San Jose, CA, USA). Reactions took place in a 96-well black-bottom plate (90 µL) at 37 °C for 30 min, during which fluorescence measurements were taken every 60 s at an excitation wavelength (λex) of 526 nm and an emission wavelength (λem) of 566 nm. All experiments were performed in triplicate.

## 3. Results and Discussion

### 3.1. Design of CRISPR/Cas12a Fluorescence Assays

As mentioned above, our target is the bla_NDM_ gene, which encodes carbapenemase. We designed our assay to detect bla_NDM-1_, but also potentially the other 31 bla_NDM_ variants (i.e., bla_NDM-2_ to bla_NDM-15_, bla_NDM-16a_, bla_NDM-16b_, and bla_NDM-17_ to bla_NDM-31_), via the following method. First, all 32 variants were aligned, which allowed us to identify a conserved region of 813 bp—denoted hereafter as bla_NDM-targeted_—that encodes the NDM enzyme. Then, within that region, we located all PAM sequences and thus all possible gRNA binding sites (Figure S1). Specifically, if bla_NDM-targeted_ is to be detected via our CRISPR/Cas12a system, a gRNA binding site should have a PAM sequence on the 5′ end of the non-complementary DNA sequence being targeted. We chose only those sequences that were identical in all 32 variants in terms of the relationships of their PAM sequences to their adjacent sequences (21 bp), and the gRNA was designed to be complementary to the adjacent sequence. Following this approach, five possible gRNA binding sites on bla_NDM-targeted_ were identified, and all five of the resulting sequences were analyzed using the Basic Local Alignment Search Tool (BLAST; U.S. National Center for Biotechnology Information, Bethesda, MD, USA). By utilizing this analytical approach, we were able to test the specificity of all five sequences and verify that our specific gRNA was suitable for use in bla_NDM_ detection.

**Figure 1:**
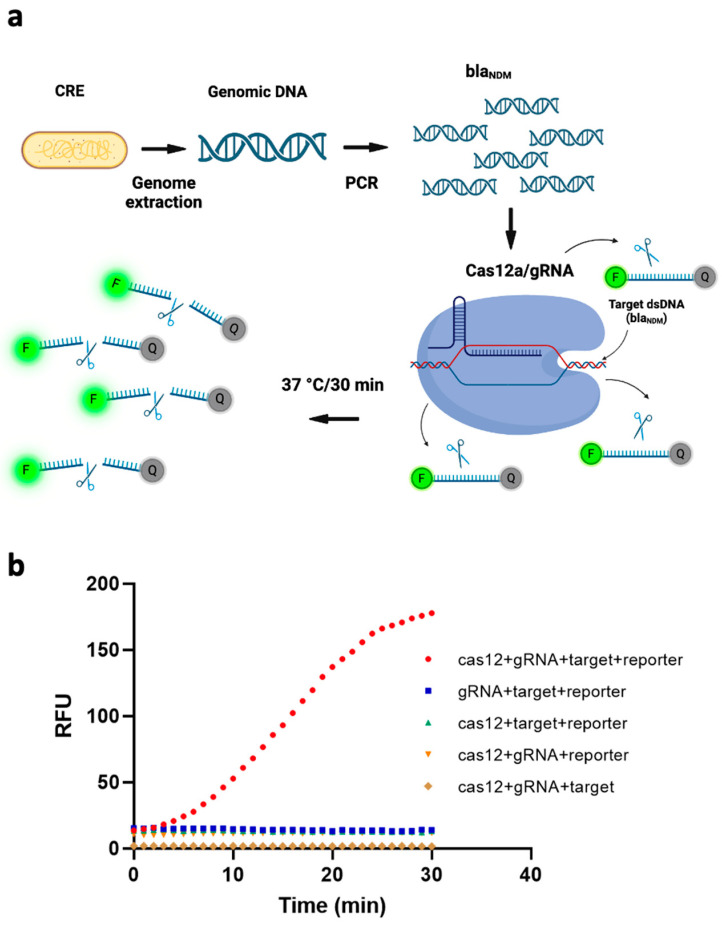
(**a**) Schematic of the working principle of a fluorescence assay for detecting bla_NDM_, based on polymerase chain reaction (PCR)-coupled clustered regularly interspaced short palindromic repeats (CRISPR) and CRISPR-associated nuclease 12a (Cas12a). The genome of carbapenem-resistant *Enterobacterales* (CRE) is extracted and bla_NDM-targeted_ is amplified via PCR. The CRISPR/Cas12a system binds to the target gene via gRNA and cleaves single-stranded DNA-fluorophore-quencher (ssDNA-FQ) reporter generating a fluorescence signal. (Image created using Biorender.com). (**b**) Four components of the CRISPR/Cas12a-based fluorescence assay—target, reporter, gRNA and Cas12—are all necessary to the assay’s functioning. RFU = relative fluorescence units.

To transduce the target gene’s recognition by our CRISPR/Cas12a/gRNA complex into sensing signals, we added a reporter ssDNA labeled with HEX fluorophore on the 5′ end and Iowa Black quencher on the 3′ end. The transduction mechanism is that, if the target sequence exists, the ssDNA FQ-reporter is cleaved (due to trans-cleavage by Cas12a), from which point the fluorophore is no longer quenched, and a fluorescent signal is emitted. Figure 1a depicts this schematically. To verify this working principle, we measured the fluorescence signal under five different conditions, i.e., without one of (1) the Cas12a protein, (2) the gRNA, (3) the target gene, or (4) the ssDNA FQ reporter; and (5) with all of them present. As can be seen from Figure 1b, all four of the conditions that included the ssDNA FQ-reporter exhibited higher fluorescence signals than the condition without it, due to spectral overlap of the fluorophore. However, the intensity of the fluorescence signal was markedly higher when all four components were present than when even one of them was missing. This further demonstrated that all the components in our system were necessary to the fluorescence assay’s functioning and that a bla_NDM-targeted_ gene can efficiently activate trans-cleavage activity of ssDNA FQ-reporters by our Cas12/gRNA system.

### 3.2. Assay Optimization

#### 3.2.1. gRNA Selection

**Figure 2:**
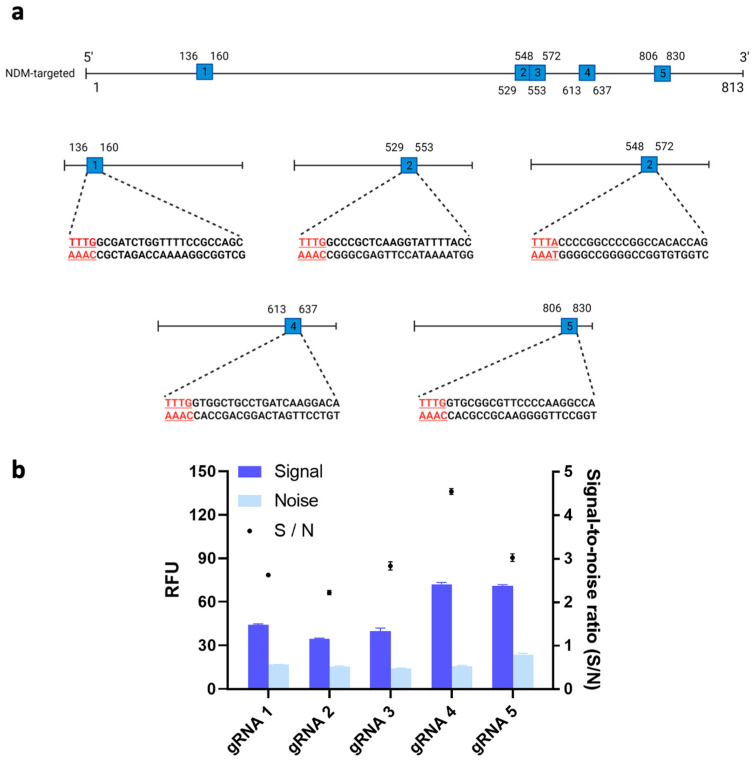
(**a**) Schematic of the guide RNA (gRNA) sites in the bla_NDM-targeted_ gene. Five sites with a protospacer-adjacent motif (PAM) sequence (i.e., TTTV) on the 5′ end of the non-complementary DNA sequence were identified. (Image created using Biorender.com). (**b**) Signal-to-noise ratio of clustered regularly interspaced short palindromic repeats (CRISPR)/CRISPR-associated nuclease 12a (Cas12a) system to detect target gene using different gRNA. 1nM of bla_NDM-targeted_ gene was added in all conditions. Error bars represent standard error of the mean (N = 3). RFU = relative fluorescence units.

It has recently been shown that the trans-cleavage ability of Cas12a could differ substantially across different types of gRNAs [22]. Thus, we tested the trans-cleavage activity and resulting fluorescent signals of Cas12a protein with five potential gRNAs and our target gene. Figure 2a shows the locations on the target gene of the five possible gRNA binding sites. We then used these five possible gRNA binding sites to design the corresponding gRNAs. Among the resulting five gRNAs, gRNA 4’s signal-to-noise (S/N) ratio, measured at 4.56, was significantly higher than the S/Ns of the other gRNAs (*p* < 0.05) (Figure 2b). Therefore, gRNA 4 was used for further optimization of our system. In line with Wu et al.’s results, the differences we observed in fluorescent signals across different loci may have been due to the complex secondary structures of the gRNA binding sites on the target gene, which could have affected the binding affinity of our Cas12a/gRNA complex [22].

#### 3.2.2. Optimization of the Amounts of Cas12a, gRNA, and ssDNA FQ-Reporter

**Figure 3:**
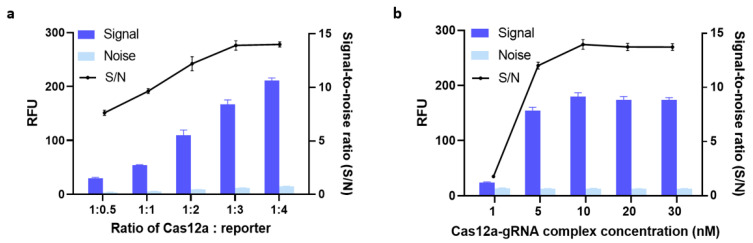
(**a**) Optimization of the Cas12a:reporter ratio. (**b**) Optimization of the concentration of a complex of clustered regularly interspaced short palindromic repeats-associated nuclease-guide RNA (Cas-gRNA). The fluorescence signal was measured after 30 min of incubation at 37 °C. 5 nM of bla_NDM-targeted_ gene was added in all conditions. Error bars represent standard error of the mean (N = 3). RFU = relative fluorescence units. (Both graphs were generated using GraphPad Prism version 9.0).

We hypothesized that using different amounts of Cas12a protein, gRNA, and reporter would profoundly affect signal output. To test this hypothesis, we first formulated five different Cas12a-to-reporter ratios and evaluated how each one was associated with signal outputs. As can be seen from Figure 3a, the fluorescent signals became stronger as these ratios increased from 1:0.5 to 1:4. The mean S/N value was almost the same when the Cas12a-to-reporter ratio was 1:3 as when it was 1:4. To achieve maximum cost-effectiveness, we therefore determined 1:3 to be the optimal ratio.

Next, we optimized the concentration of the Cas12a-gRNA complex. Previous research found no significant difference in trans-cleavage activity across Cas12a-to-gRNA ratios ranging from 1:1 to 1:3 [23]. Thus, again for cost-effectiveness reasons, we used a 1:1 ratio in our further optimization efforts. Then, the efficiency of trans-cleavage using five different concentrations of Cas12a-gRNA complex was compared. As shown in Figure 3b, the S/N ratio plateaued at around 13.88 when the concentration of Cas12a-gRNA complex was 10 nM, i.e., increasing the concentration from 10 nM to 20 nM did not increase the S/N ratio. The same increases also failed to boost the signal noticeably. Therefore, we carried on evaluating our assay with 10 nM of Cas12a-gRNA complex and a Cas12a-to-reporter ratio of 1:3 as its optimal parameters.

### 3.3. Performance Analysis of the CRISPR/Cas12a Fluorescence Assay

#### 3.3.1. Sensitivity

**Figure 4:**
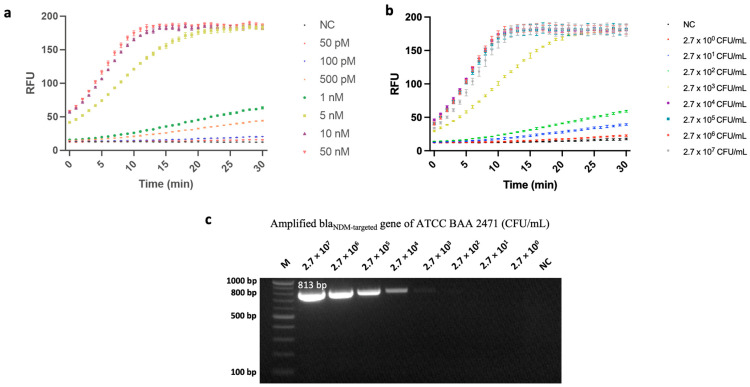
Sensitivity analysis of a fluorescence assay developed based on polymerase chain reaction (PCR)-coupled clustered regularly interspaced short palindromic repeats (CRISPR) and CRISPR-associated nuclease 12a (Cas12a). (**a**) Analysis results of the CRISPR/Cas12a fluorescence assay for concentrations of bla_NDM-targeted_ ranging from 50 pM to 50 nM. In this case, negative control (NC) refers to replacing bla_NDM-targeted_ gene with nuclease-free water. (**b**) Analysis results of the CRISPR/Cas12a fluorescence assay for concentrations of carbapenem-resistant *Enterobacterales* (CRE) ranging from 2.7 × 10^0^ CFU/mL to 2.7 × 10^7^ CFU/mL. Error bars represent standard error of the mean (N = 3). RFU = relative fluorescence units. (These graphs were generated using GraphPad Prism version 9.0). (**c**) Gel-electrophoresis image of amplified bla_NDM-targeted_ gene. Lane M: 100 bp ladder; Lanes of 2.7 × 10^7^ to 2.7 × 10^0^ CFU/mL represent the concentration of ATCC BAA 2471, NC. In this case, NC refers to replacing bacterial-genome extract with nuclease-free water.

After optimizing its parameters, we tested the fluorescence assay’s bla_NDM_-detection performance. First, amounts of bla_NDM-targeted_ gene ranging from 50 pM to 50 nM were used to evaluate its sensing performance. As shown in Figure 4a, the limit of detection (LOD) of this gene was 50 pM.

Although our developed assay is nucleic-acid-based, its main purpose is to identify the bacteria that carry AMR genes. Prior research has shown that bacteria’s genomes may affect sensors’/assays’ performance at detecting specific AMR genes within them [24]. In addition, the spread of bla_NDM_ genes is mainly facilitated by horizontal gene transfer among bacteria [25]. Thus, to be useful in real-world scenarios, our assay must be able to identify the AMR genes in bacteria in a sensitive and selective fashion. Accordingly, further analysis was conducted to evaluate its performance at detecting bacteria that carry the bla_NDM-1_ gene.

Specifically, the assay’s performance in detecting AMR bacteria was evaluated using the bla_NDM-1_ gene-carrying *E. coli* (CRE). As noted above, PCR is commonly used for detecting AMR bacteria. However, in addition to conventional PCR’s above-mentioned disadvantages, including the high labor and time costs of gel electrophoresis [12], it is uncertain in qPCR whether samples in the gray zone are positive or negative; thus, further analysis is required if valid results are to be obtained. Previous studies have shown that CRISPR/Cas12a-based assays can be more sensitive than gel-electrophoresis-based ones [26,27]. Also, Y. Chen et al. demonstrated that the former can not only address some of the drawbacks of conventional PCR/gel-electrophoresis systems but also provide a solution to qPCR’s gray-zone sample problem [28]. Therefore, we hypothesized that, if coupled with PCR, our CRISPR/Cas12a fluorescence assay would overcome the disadvantages of gel electrophoresis and therefore provide more specific and sensitive CRE sensing than PCR plus gel electrophoresis.

To test this hypothesis, we first extracted the genome of bla_NDM_-1-carrying *E. coli* with various initial concentrations (ranging from 2.7 × 10^0^ CFU/mL to 2.7 × 10^7^ CFU/mL) and amplified the bla_NDM-targeted_ gene (813 bp) via PCR. This relatively long amplicon enabled our assay to screen multiple variants of the bla_NDM_ gene. As the rate of DNA degradation is linearly proportional to the length of the amplicon [29], it would be useful for further research to explore the susceptibility of such nucleic acids to degradation. Next, our CRISPR/Cas12a sensing system was used to detect variations in genome concentrations. With a target consisting of a genomic extract of bla_NDM-1_-carrying *E. coli*, the assay attained an LOD of 2.7 × 10^0^ CFU/mL (Figure 4b). This performance surpasses that of previously published CRISPR/Cas12a-based fluorescence assays that were used to detect bacterial cells [26,30]. Additionally, Xu et al. [31] previously developed a LAMP-CRISPR-Cas12a-based lateral flow immuno-chromatographic strip for detecting CRE; however, our method’s sensitivity improved upon Xu et al.’s by approximately 5 logs. Such improvement could have resulted from variations in the two studies’ amplification and signaling methods, and/or from the comprehensive optimization we conducted.

Next, we compared the same results against gel electrophoresis, i.e., conducted such electrophoresis after PCR amplification. As can be seen from Figure 4c, after following the same amplification procedure, the PCR product could only be observed via gel electrophoresis when the bacterial concentration was higher than 2.7 × 10^2^ CFU/mL (The original, uncropped gel-electrophoresis image can be found in Figure S2). Although further optimization efforts could lead to some improvement in PCR-gel electrophoresis’ LOD, our results demonstrate that when coupled with PCR, our CRISPR/Cas12a-based method could detect PCR-amplified targets with concentrations 100-fold lower than conventional PCR-gel electrophoresis, i.e., 2.7 × 10^0^ CFU/mL vs. 2.7 × 10^2^ CFU/mL. One possible explanation for this may lie in the differences between these two methods’ fundamental mechanisms. That is, gel electrophoresis simply separates DNA fragments (i.e., PCR amplicons) based on their size, whereas our system specifically binds to the gene of interest and then generates resulting signals based on its presence. Therefore, when a nonspecific amplicon is formed during the amplification step, it is much less likely for the CRISPR/Cas12a system to generate a false-positive result because it identifies the target not by its size but by its sequence [15].

#### 3.3.2. Specificity of the CRISPR/Cas12a Fluorescence Assay

**Figure 5:**
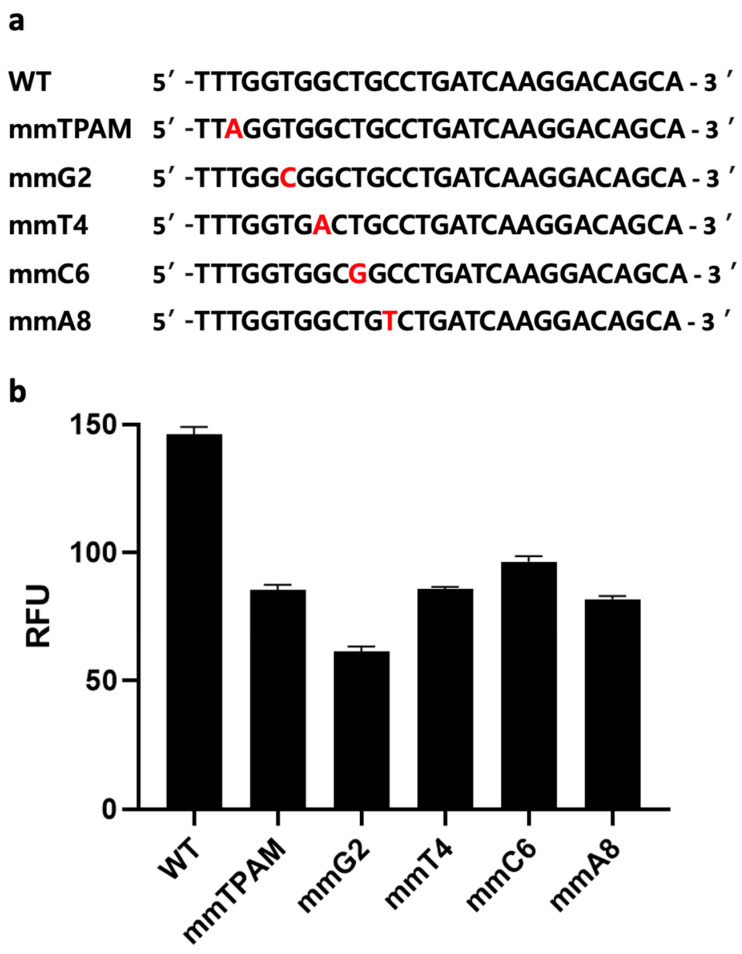
Mismatch analysis. (**a**) Target sequence with mismatches at various positions, including the third position of the protospacer adjacent motif (PAM) sequence, and guide RNA (gRNA) complementary sequences at four locations (2, 4, 6, and 8). (**b**) Evaluation of the developed clustered regularly interspaced short palindromic repeats (CRISPR) and CRISPR-associated nuclease 12a (Cas12a) fluorescence assay’s ability to distinguish single base-pair differences at various positions. Error bars represent standard error of the mean (N = 3). RFU = relative fluorescence units. (The graph was generated using GraphPad Prism version 9.0).

A distinct advantage of CRISPR-based assays over other nucleotide-detection assays (e.g., conventional PCR, qPCR, RPA, or LAMP alone) is the former’s ability to discriminate a single base-pair difference [17,32]. However, nucleic-acid amplification methods such as PCR could amplify target genes, potentially leading to higher sensitivity in sensing. Nevertheless, the above-mentioned disadvantages of conventional PCR limit its applications in the sphere of improving sensing performance. Therefore, we hypothesize that combining one of these amplification methods with a CRISPR/Cas-based one has the potential not only to improve our ability to distinguish SNP but also to eliminate false positives produced due either to mispriming or primer-dimer formation during the amplification step. For instance, when the wrong amplicon is formed, but its size is similar to a positive sample, gel electrophoresis might produce a false-positive result. However, a CRISPR/Cas-based system like ours is unlikely to generate a signal when the target gene is absent, due to a lack of gRNA binding sites. To confirm this, we introduced mismatches at five different sites on a synthetic dsDNA sequence, including the PAM sequence (Figure 5a). The five dsDNA samples with single base-pair differences were then tested using the same CRISPR/Cas12a assay described above. All five mismatch sequences exhibited significantly lower fluorescence signals compared to the original target-gene sequence (*p* < 0.05) (Figure 5b). This observation is likely due to the specific binding of Cas12a/gRNA with that gene, demonstrating the Cas12a protein’s ability to detect single base-pair differences from the original sequence. As a single-nucleotide difference can result in different AMR phenotypes, the ability to detect such differences is crucial to accurate AMR identification and epidemiology [33].

**Figure 6:**
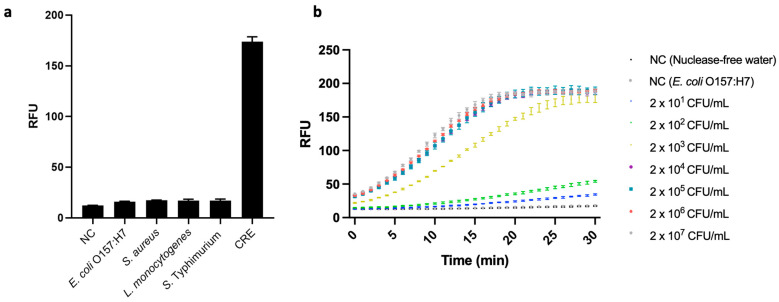
(**a**) Specificity test of the developed fluorescence assay based on polymerase chain reaction (PCR)-coupled clustered regularly interspaced short palindromic repeats (CRISPR)/CRISPR-associated nuclease 12a (Cas12a). The strains used are as follows: *Escherichia coli* O157:H7 (ATCC 43890), *Staphylococcus aureus* (ATCC 6583), *Listeria monocytogenes* (ATCC 19112), and *Salmonella enterica* serovar Typhimurium (ATCC 19585) CRE (*E. coli*, ATCC BAA 2471). Negative control (NC) refers to replacing bacterial genome extract with nuclease-free water (N = 3). (**b**) Analysis of the PCR-coupled CRISPR/Cas12a fluorescence assay for the detection of inoculated carbapenem-resistant *Enterobacterales* (CRE) in chicken samples (from 2 × 10^1^ CFU/mL to 2 × 10^7^ CFU/mL). We measured two negative controls (NCs) consisting of nuclease-free water, one without bacteria, and the other without the AMR gene (10^7^ CFU/mL of *E. coli* O157:H7). Error bars represent standard error of the mean (N = 3). RFU = relative fluorescence units. (These graphs were generated using GraphPad Prism version 9.0).

In real-world CRE detection, food and environmental samples often contain other bacteria that lack the bla_NDM_ gene. Therefore, we verified the specificity of the developed assay by using it to test for a CRE, bla_NDM-1_-carrying *E. coli*, and four other bacterial strains that did not carry bla_NDM_, i.e., *E. coli* O157:H7, *S. aureus* ATCC 6583, *L. monocytogenes* ATCC 19112, and *S.* Typhimurium ATCC 19585. Their respective sets of genomic DNA were extracted from 10^7^ CFU/mL bacterial suspensions and applied to our PCR-coupled CRISPR/Cas12a fluorescence assay. We observed high fluorescence signal only in the case of the CRE; and its value differed significantly from those of all the low-signal strains, as well as that of a negative control group (*p* < 0.05) (Figure 6a). These results demonstrate the developed assay’s excellent specificity, i.e., ability to differentiate bla_NDM_-carrying bacterial cells from others.

#### 3.3.3. Detecting bla_NDM_ in a Sample of Real Food

Although the use of carbapenems in food-producing animals is restricted in most countries, previous studies have reported a general prevalence of bla_NDM_ genes in fecal samples from livestock [34]. Other research, moreover, has found an association between bla_NDM_-containing bacteria strains in clinical samples and animal samples, indicating the inter-species transmission of these genes [35]. There are various routes by which AMR genes are transmitted between humans and animals, a major one being the food chain. For example, cross-contamination may occur due to inappropriate food handling in food-processing plants or kitchens [36]. Therefore, the feasibility of using the developed assay to monitor for AMR in the environment was evaluated with CRE-inoculated food matrices. Specifically, we used chicken because, in recent years, bla_NDM_ genes have been isolated from chicken in various parts of the world [34].

When the genomes extracted from the CRE-spiked samples were subjected to our PCR-coupled CRISPR/Cas12a fluorescence assay, the assay generated a fluorescence signal and attained an LOD of 2 × 10^1^ CFU/mL (Figure 6b), i.e., higher than in pure CRE samples. This could have been due to food matrices lowering the efficiency of bacterial-DNA extraction. However, the LOD our assay attained with chicken samples indicated that its sensitivity when detecting bla_NDM-1_ was 50 times better than that of Ong et al.’s [37] real-time PCR with the same target gene. Notably, Ong et al.’s assay was not tested against food samples, but rather against purified DNA from isolated bacterial samples.

## 4. Conclusions

In this study, a PCR-coupled CRISPR/Cas12a fluorescence assay was developed to detect CRE with bla_NDM_ genes sensitively and with high specificity. By leveraging the cis- and trans-cleavage activity of Cas12a, the developed assay specifically detected bla_NDM_ and transduced such a discovery into a fluorescence signal. To achieve excellent analytic performance, we optimized the main components of the assay, i.e., gRNA, Cas12a, and ssDNA FQ-reporter. When PCR was coupled with our CRISPR/Cas12a system, it achieved excellent analytical performance, notably including an LOD of 2.7 × 10^0^ CFU/mL of CRE-carrying bacteria. Our CRISPR/Cas12a assay was able to detect the PCR-amplified target AMR gene at 100-fold lower concentrations than conventional PCR plus gel electrophoresis could. Our assay’s performance against several common foodborne pathogens other than CRE confirmed its high specificity. Our results also demonstrate that our CRISPR/Cas system’s specific binding of gRNA to the amplicons produced by PCR enables SNP discrimination, thus overcoming another weakness of conventional PCR. We further evaluated our CRISPR/Cas12a-based assay by testing its ability to detect the target AMR gene-carrying bacteria in real food, and the results again suggested its high sensitivity.

To conclude, the developed PCR-coupled CRISPR/Cas12a fluorescence assay allows for the detection of bla_NDM_ simply and with greater sensitivity and specificity than was previously achievable via conventional PCR plus gel electrophoresis. As our assay could be conducted using equipment that is commonly available in laboratories (e.g., thermocyclers and various cost-effective alternatives to fluorometers), its barriers to access and its cost implications are both likely to be lower than those of its counterparts such as qPCR. Based on these properties, our assay can be expected to improve screening for CRE, thus potentially preventing its further spread. Moreover, it could be customized based on the testing environment. For example, depending on the facilities available, trans-cleavage activity could be transduced into a colorimetric or electrochemical signal. While adjustments may be necessary following any such alterations to the assay, it is important to bear in mind that its specificity is unlikely to be negatively impacted by them, given that it originates from the gRNA design. This convertible property further adds to our assay’s promise as a platform for mitigation of the serious global threat posed by AMR.

## Figures and Tables

**Table 1:** Polymerase chain reaction (PCR) primers for bla_NDM-targeted_ amplification, guide RNA (gRNA) synthesis primers and oligonucleotides for a clustered regularly interspaced short palindromic repeats (CRISPR) fluorescence assay.

Name	Oligonucleotide Sequence
NDM-targeted FP	5′ ATGGAATTGCCCAATATTATGCACCCGG 3′
NDM-targeted RP	5′ TCAGCGCAGCTTGTCGGCCATG 3′
gRNA synthesis FP	5′ GAAATTAATACGACTCACTATAGTAATTTCTACTAAGTGTAGAT 3′
gRNA 1 synthesis RP	5′ GCTGGCGGAAAACCAGATCGCATCTACACTTAGTAGAAATTA 3′
gRNA 2 synthesis RP	5′ GGTAAAATACCTTGAGCGGGCATCTACACTTAGTAGAAATTA 3′
gRNA 3 synthesis RP	5′ CTGGTGTGGCCGGGGCCGGGGATCTACACTTAGTAGAAATTA 3′
gRNA 4 synthesis RP	5′ TGTCCTTGATCAGGCAGCCACATCTACACTTAGTAGAAATTA 3′
gRNA 5 synthesis RP	5′ TGGCCTTGGGGAACGCCGCACATCTACACTTAGTAGAAATTA 3′
gRNA 1	5′ UAAUUUCUACUAAGUGUAGAUGCGAUCUGGUUUUCCGCCAGC 3′
gRNA 2	5′ UAAUUUCUACUAAGUGUAGAUGCCCGCUCAAGGUAUUUUACC 3′
gRNA 3	5′ UAAUUUCUACUAAGUGUAGAUCCCCGGCCCCGGCCACACCAG 3′
gRNA 4	5′ UAAUUUCUACUAAGUGUAGAUGUGGCUGCCUGAUCAAGGACA 3′
gRNA 5	5′ UAAUUUCUACUAAGUGUAGAUGUGCGGCGUUCCCCAAGGCCA 3′
ssDNA-FQ (HEX labeled)	5′ HEX-TTTTTTTTTT-IABkFQ 3′
WT (gRNA4) *	5′ TTTGGTGGCTGCCTGATCAAGGACAGCA 3′
mmTPAM *	5′ TTAGGTGGCTGCCTGATCAAGGACAGCA 3′
mmG2 *	5′ TTTGGCGGCTGCCTGATCAAGGACAGCA 3′
mmT4 *	5′ TTTGGTGACTGCCTGATCAAGGACAGCA 3′
mmC6 *	5′ TTTGGTGGCGGCCTGATCAAGGACAGCA 3′

* dsDNA; NDM: New Delhi metallo-β-lactamase; FP: forward primer; RP: reverse primer; ssDNA-FQ: single-stranded DNA-fluorophore quencher.

## Data Availability

Data will be made available on request.

## Supplementary Materials

The following supporting information can be downloaded at: https://www.mdpi.com/article/10.3390/bios14040194/s1, Figure S1: Alignments of bla_NDM_ variants and identification of the binding sites for our guide ribonucleic acid (gRNA) variants; Figure S2: Original gel-electrophoresis image of amplified bla_NDM-targeted_ gene.
