# Supplementary material for: A CRISPR/Cas12a-Based System for Sensitive Detection of Antimicrobial-Resistant Genes in Carbapenem-Resistant Enterobacterales
Source: Biosensors (Basel). 2024 Apr 16;14(4):194. doi: 10.3390/bios14040194 (PMC11048238; doi:10.3390/bios14040194)
Supplement: Supplementary file 1 [file biosensors-14-00194-s001.zip › biosensors-2906142-supplementary.pdf]

Supplementary Material

a

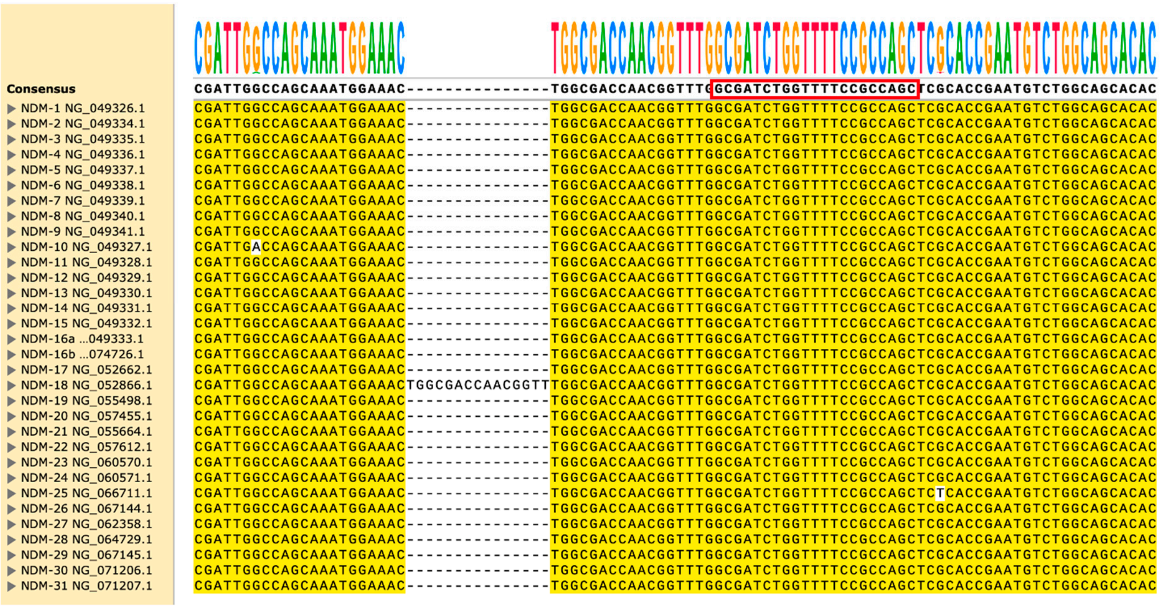

b

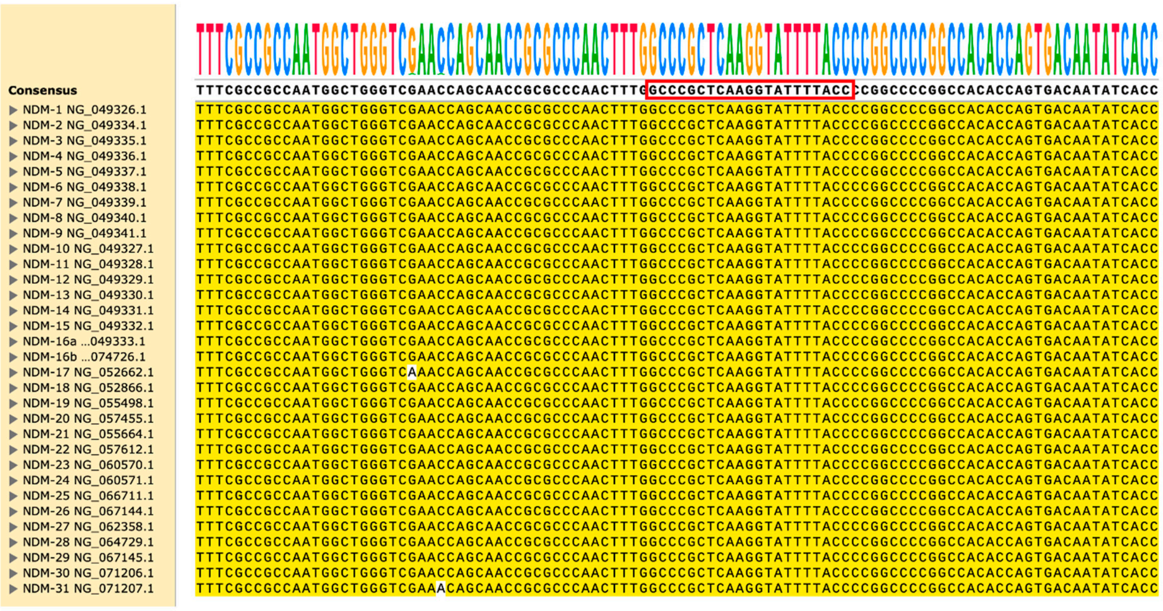

c

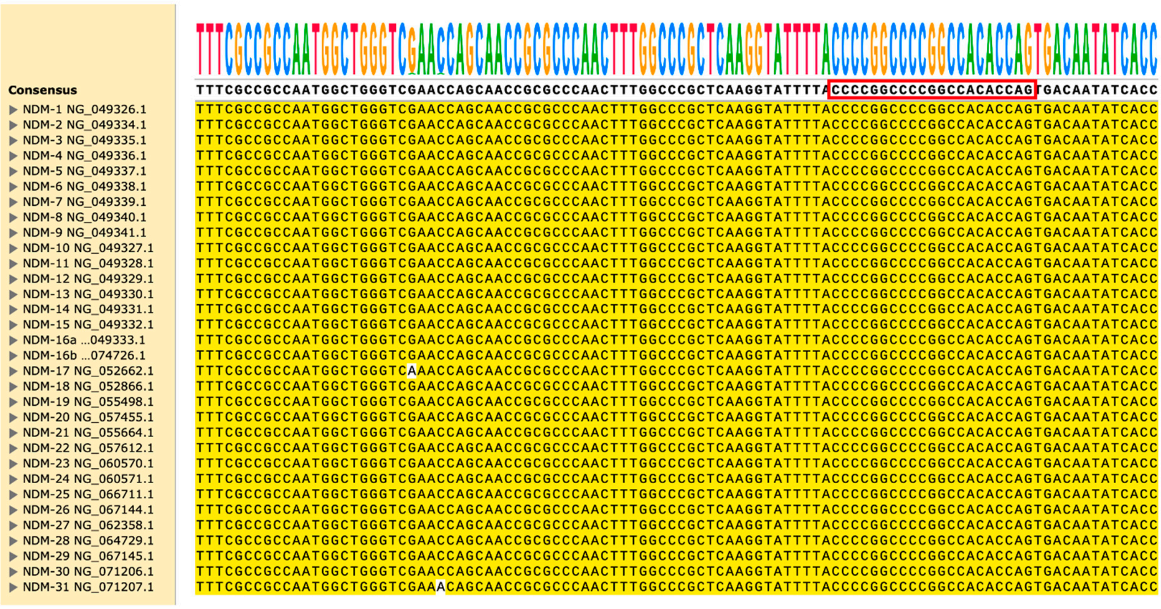
Consensus
TTTCGCCGCCAATGGCTGGGTCGAACCAGCAACCGCGCCCAACTTTGGCCCGCTCAAGGTATTTTACCCCGGCCCCGGCCACACCAGTGACAATATCACC
NDM-1 NG_049326.1
NDM-2 NG_049334.1
NDM-3 NG_049335.1
NDM-4 NG_049336.1
NDM-5 NG_049337.1
NDM-6 NG_049338.1
NDM-7 NG_049339.1
NDM-8 NG_049340.1
NDM-9 NG_049341.1
NDM-10 NG_049327.1
NDM-11 NG_049328.1
NDM-12 NG_049329.1
NDM-13 NG_049330.1
NDM-14 NG_049331.1
NDM-15 NG_049332.1
NDM-16a ...049333.1
NDM-16b ...074726.1
NDM-17 NG_052662.1
NDM-18 NG_052866.1
NDM-19 NG_055498.1
NDM-20 NG_057455.1
NDM-21 NG_055664.1
NDM-22 NG_057612.1
NDM-23 NG_060570.1
NDM-24 NG_060571.1
NDM-25 NG_066711.1
NDM-26 NG_067144.1
NDM-27 NG_062358.1
NDM-28 NG_064729.1
NDM-29 NG_067145.1
NDM-30 NG_071206.1
NDM-31 NG_071207.1

d

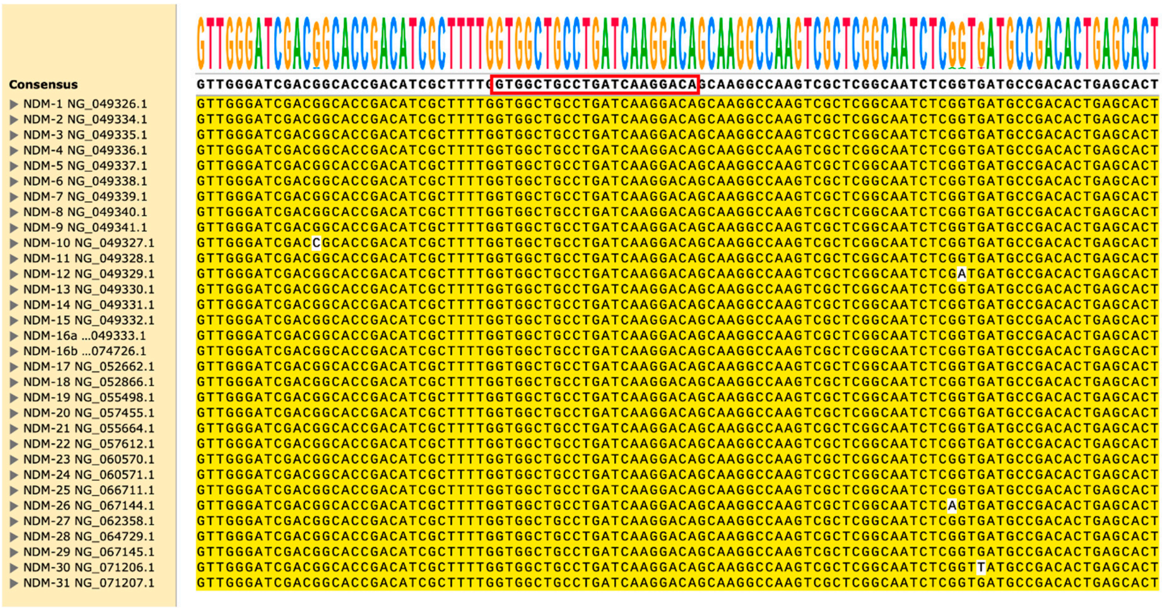
Consensus
GTTGGGATCGACGGCACCGACATCGCTTTTGGTGGCTGCCTGATCAAGGACAGCAAGGCCAAGTCGCTCGGCAATCTCGGTGATGCCGACACTGAGCACT
NDM-1 NG_049326.1
NDM-2 NG_049334.1
NDM-3 NG_049335.1
NDM-4 NG_049336.1
NDM-5 NG_049337.1
NDM-6 NG_049338.1
NDM-7 NG_049339.1
NDM-8 NG_049340.1
NDM-9 NG_049341.1
NDM-10 NG_049327.1
NDM-11 NG_049328.1
NDM-12 NG_049329.1
NDM-13 NG_049330.1
NDM-14 NG_049331.1
NDM-15 NG_049332.1
NDM-16a ...049333.1
NDM-16b ...074726.1
NDM-17 NG_052662.1
NDM-18 NG_052866.1
NDM-19 NG_055498.1
NDM-20 NG_057455.1
NDM-21 NG_055664.1
NDM-22 NG_057612.1
NDM-23 NG_060570.1
NDM-24 NG_060571.1
NDM-25 NG_066711.1
NDM-26 NG_067144.1
NDM-27 NG_062358.1
NDM-28 NG_064729.1
NDM-29 NG_067145.1
NDM-30 NG_071206.1
NDM-31 NG_071207.1

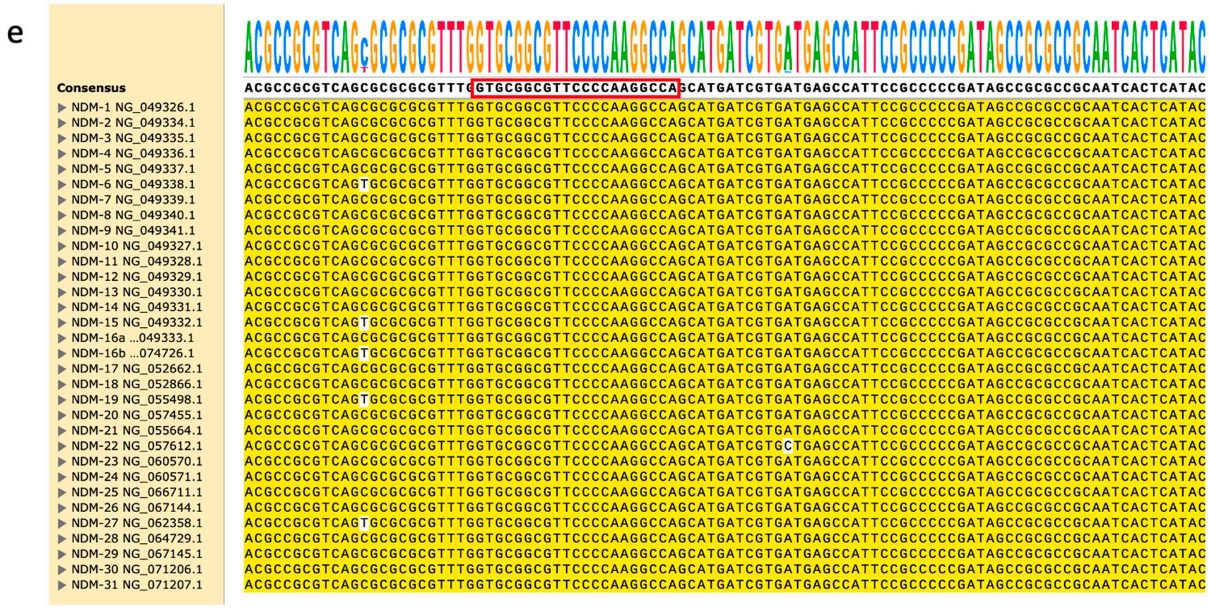

Figure S1. Alignments of bla_{NDM} variants and identification of the binding sites for our guide ribonucleic acid (gRNA) variants. All 32 bla_{NDM} variants (i.e., bla_{NDM-1} to bla_{NDM-15}, $bla_{NDM-16a}$, $bla_{NDM-16b}$, and bla_{NDM-17} to bla_{NDM-31}) were aligned, and the gRNA binding sites are marked with red boxes for gRNA 1 through gRNA 5 in panels (a)-(e), respectively. All alignment results were obtained via Snapgene, and other gene information was from the NCBI database.

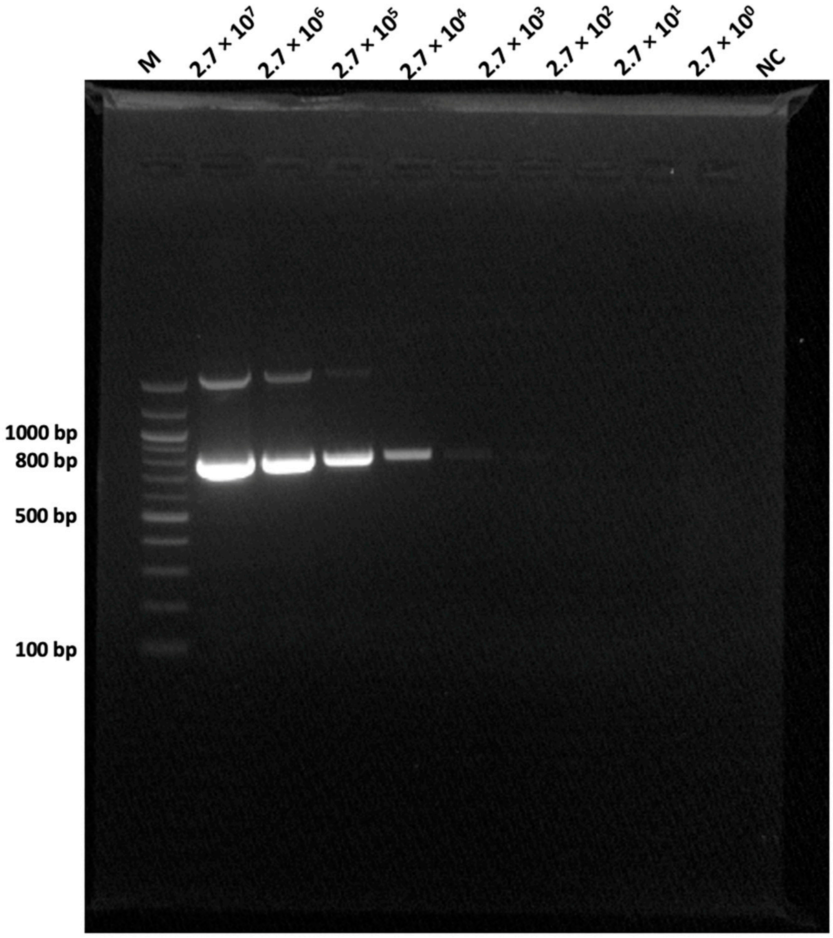

Figure S2. Original gel-electrophoresis image of amplified $bla_{NDM\text{-}targeted}$ gene. Lane M: 100 bp ladder; Lanes 1-9: ATCC BAA 2471 2.7 × 10^7 to 2.7 × 10^0 CFU/mL, NC. In this case, NC refers to replacing bacterial-genome extract with nuclease-free water
